# Disease-driven loss of inactive *HSD17B13* isoforms enhances enzymatic output in MASH and counters protective rs72613567:TA variant

**DOI:** 10.1016/j.jhepr.2026.101793

**Published:** 2026-02-23

**Authors:** John Min, Mulugeta Seneshaw, Faridoddin Mirshahi, Hae-Ki Min, Arun J. Sanyal

**Affiliations:** 1 Division of Gastroenterology, Hepatology and Nutrition, Virginia Commonwealth University, Richmond, VA, USA; 2 Dept. of Pharmacology, University of Virginia School of Medicine, Charlottesville, VA, USA

**Keywords:** *HSD17B13*, alternative splicing, isoform regulation, metabolic dysfunction–associated steatohepatitis (MASH), metabolic dysfunction–associated steatotic liver disease (MASLD), noncoding RNA, RNA secondary structure, rs72613567:TA variant, RNA interference

## Abstract

**Background & Aims:**

We investigated whether liver disease alters *HSD17B13* isoform expression, identifying selective loss of exon 2-skipped variants and uncovering variant B as a structured, noncoding RNA with silencing potential.

**Methods:**

Human liver samples from lean control (n = 6), metabolic dysfunction-associated steatotic liver (MASL, n = 8), and metabolic dysfunction-associated steatohepatitis (MASH, n = 8) participants were analyzed by isoform-specific reverse-transcription PCR and quantitative PCR. HepG2 cells were transfected with *HSD17B13* variant A or B constructs. RNA expression, protein production, RNase sensitivity, and RNA structural conformations (RNAfold) were evaluated. Functional effects were tested under oleic acid–induced lipotoxic stress.

**Results:**

We observed a selective reduction in exon 2–skipped *HSD17B13* isoforms (variants B and G) in both human MASL (∼63% *vs*. lean control; *p* <0.01) and MASH (∼93% *vs*. lean control; *p* <0.001), independent of rs72613567:TA genotype. Notably, variant B nearly abolished endogenous *HSD17B13* expression in HepG2 cells (∼99% reduction; *p* <0.001) without generating detectable protein. RNase III sensitivity assays and RNAfold modeling revealed stable, duplex-rich RNA structures, supporting a noncoding regulatory role for these isoforms under oleic acid–induced lipotoxic stress.

**Conclusions:**

Restoration of exon 2–skipped *HSD17B13* isoforms, particularly variant B, may offer a genotype-independent therapeutic strategy for MASH by mimicking protective effects through structured RNA-mediated suppression of enzymatic HSD17B13 activity.

**Impact and implications:**

These findings support a dual mechanism of HSD17B13 regulation in liver disease through genotype-mediated transcript suppression and disease-driven isoform imbalance. Therapeutic re-expression of exon 2-skipped isoforms, particularly variant B, may offer a novel strategy to replicate the protective effect of the TA allele without enzymatic inhibition.

## Highlights

• Exon 2–skipped *HSD17B13* isoforms are selectively lost in MASL and MASH.
• Variant B acts as a noncoding RNA that suppresses endogenous *HSD17B13.*
• RNase III sensitivity and RNAfold modeling reveal structured duplex RNA in variant B.
• Restoring exon 2-skipped isoforms may offer a therapeutic approach in MASH.

## Graphical abstract

**Figure:**
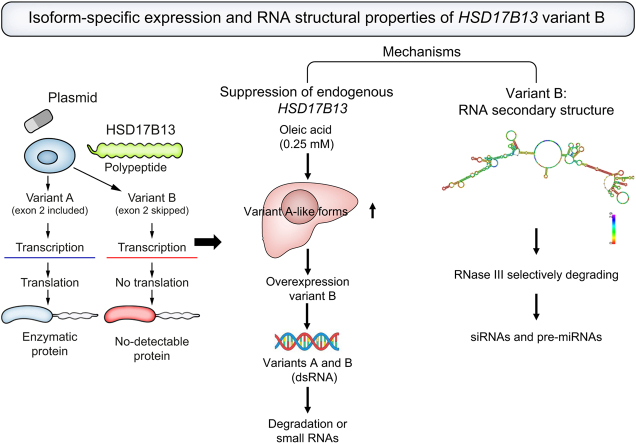

## Introduction

Hydroxysteroid 17-beta dehydrogenase *13* (*HSD17B13*) encodes a hepatic lipid droplet–associated short-chain dehydrogenase implicated in the pathogenesis of metabolic dysfunction-associated steatotic liver disease (MASLD) and its progressive subtype, metabolic dysfunction-associated steatohepatitis (MASH).[1], [2], [3] A common splice-altering variant, rs72613567:TA, introduces an alternative acceptor site upstream of exon 2, resulting in exon 2 skipping and the formation of truncated or inactive *HSD17B13* transcripts.^4^ This variant has been robustly associated with protection from chronic liver diseases^4^^,^^5^ and is now being translated into HSD17B13 silencing as a way to treat MASH.^6^ While the protective effect of the TA allele is thought to arise from reduced enzymatic activity, the specific transcript isoforms affected, particularly in the context of liver disease, have not been comprehensively characterized.

*HSD17B13* generates multiple alternatively spliced isoforms, including exon 2–included variants (A, C, E, F) with enzymatic potential and exon 2–skipped variants (B, G) lacking catalytic activity.^7^ All individuals express several splice forms, but it remains unclear whether MASLD and MASH alter isoform distribution independent of genotype or whether such disease-related shifts mimic or oppose those seen in rs72613567:TA carriers.

Previous studies examined how the rs72613567:TA genotype influences *HSD17B13* isoform expression.^4^ Among eight annotated transcripts, both exon 2–included and exon 2–skipped isoforms were progressively reduced in T/TA and TA/TA carriers compared to T/T homozygotes.^2^^,^^4^ Transcript D was increased in TA/TA individuals, suggesting compensatory splicing, while transcript H remained stable. These findings indicate that the protective TA allele broadly decreases *HSD17B13* transcript abundance rather than selectively promoting exon 2 skipping, leading to loss of both enzymatic and non-enzymatic isoforms. The molecular mechanisms remain undefined, limiting understanding of disease modulation and presenting opportunities for therapeutic exploration.

In this study, we analyzed hepatic *HSD17B13* isoform expression across controls, MASL, and MASH, revealing a progressive loss of variant B. Overexpression of variant B in HepG2 cells formed RNA duplexes leading to degradation and reduced protein levels. These results support a dual regulatory model and highlight exon 2-skipping correction as a potential therapeutic approach for MASH.

## Materials and methods

Detailed materials and methods are provided in the supplementary information.

## Results

### Exon 2–skipped *HSD17B13* transcripts are selectively suppressed in MASL and MASH, independent of rs72613567:TA genotype

**Fig. 1:**
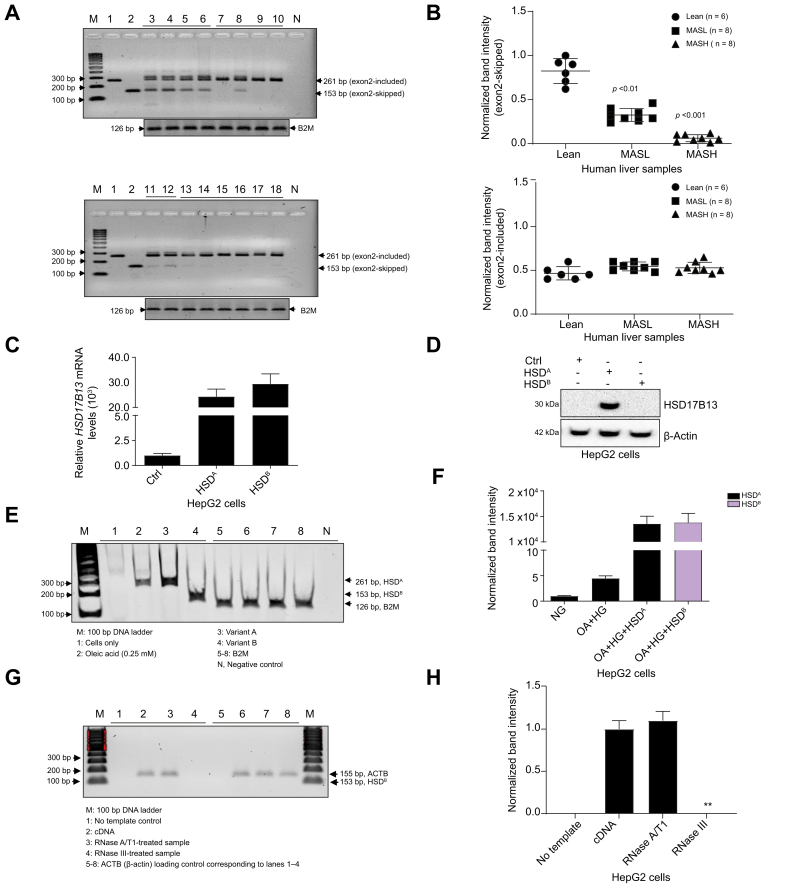
Exon 2-skipped *HSD17B13* isoforms are selectively suppressed in MASH and exhibit regulatory, non-enzymatic activity. (A) Semi-quantitative RT-PCR of HSD17B13 in human liver samples: control plasmids (lanes 1–2), lean control (3-6), MASL (7-10), and MASH (13–18). Primers flanking exon 2 distinguished variant A (261 bp) from variant B (153 bp); B2M (126 bp) was used as a control. (B) Quantification of *HSD17B13* exon 2-skipped transcript levels (upper panel) and exon 2-included transcripts (lower panel) by qPCR in lean control, MASL, and MASH liver samples, showing disease-associated reduction in non-enzymatic isoforms. (C) qPCR analysis of total *HSD17B13* mRNA in HepG2 cells transfected with variant A or variant B constructs. (D) Immunoblot analysis of HSD17B13 protein expression in HepG2 cells following overexpression of variant A or variant B. Variant A produces detectable protein; variant B does not. (E) Semi-quantitative RT-PCR assessing endogenous *HSD17B13* transcript levels after oleic acid treatment (0.25 mM) with or without expression of variant A or variant B. *B2M* was included as a loading control. (F) Densitometric quantification of 261 bp (variant A-like) and 153 bp (variant B-like) bands from (E), normalized to *B2M*. Expression of variant B suppressed oleic acid–induced *HSD17B13* expression. (G) RNase sensitivity assay of total RNA from HepG2 cells transfected with plasmid expressing variant B. Samples were digested with RNase A/T1 (ssRNA-specific) or RNase III (dsRNA-specific), followed by RT-PCR using exon 2–flanking primers to detect variant B (153 bp). *ACTB* (155 bp) served as the internal control. (H) Quantification of normalized band intensity following RNase digestion. Variant B exhibited marked sensitivity to RNase III but not to RNase A/T1, consistent with the presence of structured or dsRNA elements. dsRNA, double-stranded RNA; MASL, metabolic-associated steatosis with lipid accumulation; MASH, metabolic dysfunction–associated steatohepatitis; RNase, ribonuclease; RT-PCR, reverse transcription PCR; ssRNA, single-stranded RNA.

To investigate whether *HSD17B13* isoform expression is also altered in a disease-dependent context, we performed isoform-specific reverse-transcription PCR (RT-PCR) and quantitative PCR on liver tissues from lean control, MASL, and MASH participants (Fig. 1A,B). Detailed patient characteristics are provided in Table S1, with a brief summary of age, sex, BMI, indication for biopsy, fibrosis stage, and key biochemical parameters included here. In control livers, both exon 2-included (261 bp; transcripts A, C–F) and exon 2-skipped (153 bp; transcripts B, G) isoforms were abundantly expressed. In contrast, exon 2-skipped variants B and G were markedly reduced in MASL and MASH, while exon 2-included isoforms remained stably expressed or modestly increased. This pattern was consistent across samples, including those lacking the rs72613567:TA allele, indicating that suppression of exon 2-skipped isoforms is disease-driven and genotype independent. Together, these findings demonstrate that although genetic and disease-associated mechanisms alter *HSD17B13* splicing, they do so in distinct and non-redundant ways – one favoring transcript loss, the other favoring isoform imbalance.

### Isoform-specific expression and RNA structural properties of *HSD17B13* variant B

HepG2 cells were transfected with plasmids expressing *HSD17B13* variant A (exon 2–included, active) or variant B (exon 2–skipped, inactive) to assess functional differences. Variant A overexpression led to increased *HSD17B1*3 mRNA and protein expression, consistent with efficient translation and enzymatic function.^4^^,^^5^ In contrast, variant B led to high mRNA levels but no detectable protein, suggesting translational repression, transcript instability, or absence of a functional open reading frame. These findings support a model in which variant B acts through non-coding or RNA-based regulatory mechanisms rather than enzymatic activity (Fig. 1C,D).

To examine whether *HSD17B13* isoforms influence endogenous gene expression under metabolic stress, HepG2 cells were treated with oleic acid (0.25 mM), a physiologic inducer of lipogenesis. As expected, oleic acid alone increased *HSD17B13* transcript levels, consistent with its role in activating lipid droplet-associated gene programs.^8^ Overexpression of variant A further enhanced this response, suggesting additive transcriptional activation or stabilization of endogenous mRNA. In contrast, cells overexpressing variant B exhibited marked suppression of oleic acid-induced endogenous *HSD17B13* variant A gene expression (Fig. 1E,F). Semi-quantitative RT-PCR and densitometry showed reduced 261 bp transcript level (variant A) in variant B–transfected cells, despite stable B2M control (Fig. 1E), suggesting post-transcriptional silencing of variant A via RNA duplex formation or miRNA-like mechanisms. This autoregulatory function may act as a negative feedback mechanism to limit excessive HSD17B13 activity during lipotoxic stress, mirroring the protective downregulation observed in rs72613567:TA carriers.^4^^,^^9^

To investigate whether *HSD17B13* transcript isoforms differ in RNA structural characteristics, we performed ribonuclease (RNase) sensitivity assays using total RNA isolated from HepG2 cells transfected with plasmid encoding variant B (153 bp; exon 2-skipped). Two enzymes with distinct substrate specificities were used: RNase A/T1, which cleaves single-stranded RNA, and RNase III, which selectively degrades double-stranded RNA (dsRNA), including stem-loops and duplex regions (Fig. 1G).^4^ Following enzymatic digestion, semi-quantitative RT-PCR was performed using primers flanking exon 2 to simultaneously detect the isoform, while *ACTB* (155 bp) served as the internal normalization control. After RNase A/T1 treatment, variant B remained largely intact, suggesting limited single-stranded exposure under native conditions. In contrast, RNase III treatment resulted in substantial degradation of the isoform, indicating the presence of stable secondary structures such as stem-loops or duplex regions that render these transcripts susceptible to dsRNA-specific cleavage (Fig. 1H). Notably, variant B was particularly sensitive to RNase III, implying a higher degree of intramolecular base-pairing and compact folding in the exon 2–skipped isoform. These findings are consistent with computational RNA secondary structure predictions (Fig. S1), which showed that the variant B transcript folds into a compact, duplex-rich conformation with a minimum free energy value below –220 kcal/mol. These stability data align with those observed in regulatory noncoding RNAs.^10^

These structured RNA configurations are known to mediate post-transcriptional silencing, promote mRNA decay, and facilitate recruitment of RNA-binding proteins or RNA interference machinery.^9^^,^^10^ These structural features may underlie variant B’s ability to suppress endogenous *HSD17B13* expression under metabolic stress (Fig. 1F), suggesting that variant B may function not via protein translation, but as a regulatory RNA. This noncoding behavior could mirror the transcriptomic suppression observed in rs72613567:TA carriers, offering a potential mechanistic link between isoform architecture and genetic protection.[11], [12], [13]

## Discussion

In this study, we identify a disease-driven shift in *HSD17B13* isoform usage that opposes the protective splicing pattern linked to the rs72613567:TA variant. While this germline insertion promotes exon 2 skipping and reduces enzymatic isoforms,^4^^,^^7^ our transcript-level analysis shows that progression from healthy liver to MASL and MASH selectively suppresses exon 2-skipped transcripts, particularly variants B and G. In contrast to prior findings,^4^ where TA carriers exhibited higher variant G levels, our MASH samples showed reduced expression of both B and G. These results suggest that liver disease itself modulates *HSD17B13* splicing and alters isoform balance. The observed isoform differences may be partially driven by obesity-related metabolic factors, but the consistent loss of exon 2-skipped isoforms with worsening disease suggests a role for liver injury severity that future weight-matched cohorts must clarify. Together with prior studies, these findings reveal two mechanisms by which HSD17B13 may influence MASLD progression. In genetically protected individuals, the rs72613567:TA allele broadly reduces *HSD17B13* transcript abundance, affecting both enzymatic (variant A) and non-enzymatic isoforms (variants B and G) through splice-altering mechanisms that yield unstable transcripts.^4^^,^^7^ In contrast, liver disease progression selectively depletes non-enzymatic exon 2-skipped isoforms while preserving or increasing exon 2-included variants, shifting expression toward catalytically active forms that may exacerbate hepatocellular stress, inflammation, and fibrosis in MASH.^5^^,^^14^

Functional studies in HepG2 cells revealed distinct roles for *HSD17B13* isoforms. Variant A produced both mRNA and protein, confirming enzymatic activity, whereas variant B increased mRNA without detectable protein and suppressed endogenous *HSD17B13* under lipogenic stress, suggesting a noncoding regulatory role. RNase assays showed that variant B was sensitive to RNase III, exhibiting a higher degree of dsRNA structure and greater silencing potential. These findings indicate that structured RNA elements are a defining feature of *HSD17B13* isoforms, particularly variant B, which may function as a regulatory RNA. Variant B may engage RNA interference machinery or dsRNA–binding proteins such as Dicer and Argonaute.^15^^,^^16^ Although not directly tested, RNAfold and Biopython modeling indicated that variant B forms thermodynamically stable secondary structures, with a minimum free energy of –223.80 kcal/mol. These include hairpins, stem-loops, and duplexes exceeding 21 nt (Fig. S1A), characteristic of endogenous regulatory RNAs such as long noncoding RNAs or truncated mRNA isoforms.^10^^,^^12^ Predicted small-interfering RNA- and pre-miRNA-like regions are summarized in Table S2. The structured nature of variant B may promote transcript silencing or cis/trans inhibition of full-length HSD17B13. Its suppression under lipotoxic stress likely involves dsRNA complex formation and RNA interference-like degradation,^17^ potentially mimicking the rs72613567:TA protective effect, which reduces HSD17B13 enzymatic activity through exon 2 skipping and transcript destabilization. Validation in additional liver cell models and independent human cohorts will be necessary to confirm the generalizability and translational relevance of these findings. These findings also suggest that therapeutic restoration of exon 2–skipped HSD17B13 isoforms – using splice-modulating oligonucleotides or RNA-stabilizing approaches – may offer a genotype-independent molecular strategy for MASH. Further preclinical evaluation of isoform-augmentation therapies will be essential to determine feasibility and therapeutic potential.

In summary, our findings thus support a dual regulatory model:^1^ the TA allele reduces total *HSD17B13* expression, offering genetic protection;^2^ MASH progression selectively depletes noncoding, exon 2–skipped isoforms, favoring enzymatic variants. Restoring variant B may mimic TA-mediated protection through adeno-associated virus delivery, RNA mimetics, or splicing modulation. Future studies will further evaluate^1^ the mechanisms by which *HSD17B13* gene variants modulate their own expression,^2^ specific mechanisms of disease-driven isoform imbalance and^3^ *in vitro* and *in vivo* therapeutic interventions using restoration of exon 2 transcription.

## Abbreviations

dsRNA, double-stranded RNA; HSD17B13, hydroxysteroid 17-beta dehydrogenase 13; MASLD, metabolic dysfunction–associated steatotic liver disease; MASL, metabolic-associated steatosis with lipid accumulation; MASH, metabolic dysfunction–associated steatohepatitis; RNase, ribonuclease; RT-PCR, reverse transcription PCR.

## Authors’ contributions

Conceived the project: AJS, HK, JM. Designed experiments: all authors. Performed experiments, collected data, and analyzed data: JM, MS, FM, HM. Drafted the manuscript: AJS, HM. Edited and finalized the manuscript: all authors. Secured funding: AJS.

## Declaration of generative AI and AI-assisted technologies in the writing process

Generative AI was used to assist with language editing and figure formatting. All scientific content and interpretations were produced and verified by the authors.

## Financial support

This work was supported by intramural funds from the Stravitz-Sanyal Institute for Liver Disease and Metabolic Health. The Institute did not have any role in the conduct of the trial or its interpretation.

## Conflicts of interest

AJS: AJS has stock options in Tiziana, Rivus, Durect, NorthSea. He has served as a paid consultant to Intercept, Genfit, Boehringer Ingelhiem, Eli Lilly, Novo Nordisk, Glaxo Smith Kline, Madrigal, Amgen, Genentech, Merck, Zydus, Astra Zeneca, Alnylam, Regeneron, Altimmune, Surrozen, Poxel, Hanmi, Akero Therapeutics, Boston Pharma, 89 Bio, Pliant, Chemomab, Salix, TARGET-MASH, Path AI, Histoindex. His institution receives funding from Avant Sante for consultation with him and has received grants from Novo Nordisk, Hanmi, 89 Bio, Madrigal, Gilead, Akero, Merck, Takeda, Salix, Intercept and Genfit. He receives royalties from Elsevier and Wolter Kluwers.

Please refer to the accompanying ICMJE disclosure forms for further details.
