# Supplementary material for: Disease-driven loss of inactive HSD17B13 isoforms enhances enzymatic output in MASH and counters protective rs72613567:TA variant
Source: JHEP Rep. 2026 Feb 23;8(5):101793. doi: 10.1016/j.jhepr.2026.101793 (PMC13101278; doi:10.1016/j.jhepr.2026.101793)
Supplement: Multimedia component 1 [file mmc1.pdf]

Disease-driven loss of inactive *HSD17B13* isoforms enhances enzymatic output in MASH and counters protective rs72613567:TA variant

John Min, Mulugeta Seneshaw, Faridoddin Mirshahi, Hae-Ki Min, Arun J. Sanyal

Table of contents

Materials and methods……………………………………………………………..………2

Supplementary tables………..……………………………………………….……….…..5

Supplementary figure…………………………………………………………….……..7

Original Gel blots and Immunoblot ….…………………………….………………..……8

Supplementary references...…………………………….……………....…….……12

Materials and Methods

Human liver tissue

Human liver tissues were obtained from participants undergoing bariatric surgery or clinically indicated liver biopsy under IRB-approved protocols (VCU IRB No. 1960). The nonalcoholic nature of liver disease and the clinical, demographic, and laboratory characteristics of the cohort have been described previously(1)(**Supplementary Table1**). Liver samples were classified as lean control, metabolic dysfunction–associated fatty liver (MASL), or metabolic dysfunction–associated steatohepatitis (MASH) based on histological scoring according to the Nonalcoholic Steatohepatitis Clinical Research Network (NASH CRN) criteria(2). All participants provided written informed consent.

Cell culture and transfection

HepG2 cells (ATCC) were maintained in low-glucose DMEM supplemented with 10% fetal bovine serum (FBS) and 1% penicillin–streptomycin at 37 °C in a 5% CO_2 incubator. Self-complementary AAV8 plasmids encoding *HSD17B13* Variant A (scAAV8.TBG-HSD17B13-A) and Variant B (scAAV8.TBG-HSD17B13-B), each under the control of a hepatocyte-specific thyroxine-binding globulin (TBG) promoter, were obtained from the Gene Therapy Center at the University of Massachusetts Medical School. Cells were seeded at a density of 3×10^5 cells per well in 6-well plates and cultured to ~70~80% confluency. Transfection was performed using 1.5 µg of either *HSD17B13* Variant A or Variant B plasmid with Lipofectamine 3000 (Thermo Fisher Scientific, Cat# L3000008), according to the manufacturer's instructions. An empty vector (pcDNA3.1) was used as a control.

RNA extraction and RT-PCR/qPCR

Total RNA was extracted from liver tissue and HepG2 cells using TRIzol reagent (Invitrogen) and reverse transcribed with SuperScript IV reverse transcriptase (Thermo Fisher Scientific). Isoform-specific primers (**Supplementary Table 2**) were designed to distinguish between exon 2-included and exon 2-skipped *HSD17B13* transcripts. Semi-quantitative RT-PCR products were electrophoretically separated on 2% agarose or 8% polyacrylamide gels, stained with ethidium bromide, and visualized using a FluorChem M imaging system (ProteinSimple, San Jose, CA).

Densitometric quantification of band intensity was performed using ImageJ software. Quantitative PCR was performed using SYBR Green Master Mix (Applied Biosystems), with β2-microglobulin (B2M) or β-actin (*ACTB*) as internal controls. Relative transcript levels were calculated using the 2^–ΔΔCt method.

Immunoblotting

Whole-cell lysates were prepared using RIPA buffer supplemented with protease inhibitors. Protein concentration was quantified using the BCA assay (Pierce). Equal amounts of protein (20–30 µg) were separated by SDS–PAGE and transferred to PVDF membranes. Membranes were probed with primary antibodies against HSD17B13 (Abcam) and β-actin (ACTB; Cell Signaling Technology) as a loading control, followed by HRP-conjugated secondary antibodies. Immunoreactive bands were visualized using a FluorChem M imaging system (ProteinSimple, San Jose, CA). Densitometric analysis was performed with ImageJ software, and protein expression levels were normalized to β-actin.

RNase Digestion and RNA Structural Prediction Assay

To evaluate isoform-specific RNase sensitivity, HepG2 cells were transfected with self-complementary AAV8 vectors encoding *HSD17B13* Variant A (HSD^A) or Variant B (HSD^B), under the control of the hepatocyte-specific TBG promoter. Total RNA was extracted using TRIzol reagent (Invitrogen), quantified, and incubated with either RNase A/T1 Mix (Thermo Fisher Scientific, Cat# EN0551) or RNase III (New England Biolabs, Cat# M0245S) at 37 °C for 30 minutes under native conditions. RNase A/T1 selectively digests single-stranded RNA (ssRNA), whereas RNase III targets double-stranded RNA (dsRNA), enabling interrogation of isoform-specific RNA structural features. Following digestion, cDNA was synthesized using M-MLV reverse transcriptase (Invitrogen) and oligo(dT) primers. RT-PCR was performed using variant-specific primers flanking exon 2 to distinguish HSD^A (261 bp) and HSD^B (153 bp) isoforms. β-actin (*ACTB*) served as the internal control. To complement the in vitro assay, RNA secondary structure predictions were performed using the ViennaRNA package RNAfold (v2.5.1)(3). Full-length ORF sequence of HSD17B13 Variant B was submitted in FASTA format under default thermodynamic conditions (37 °C, no constraints). Minimum free energy (MFE) structures were calculated and visualized using RNAplot, a component of the ViennaRNA package. Resulting

structures showed MFE value of –223.80 kcal/mol for Variant B. Structural features, including hairpins, stem-loops, and internal duplex regions ≥21 nt, were annotated using Biopython (v1.81)(4). These predicted conformation (see **Supplemental Figure 1**) support the presence of double-stranded elements consistent with the RNase III digestion profile observed for Variant B, in agreement with prior observations that MFE modeling reliably predicts RNA folding and RNase susceptibility(5). Predicted siRNA- and pre-miRNA-like regions are summarized in **Supplementary Table 3**.

Statistical analysis

All experiments were performed with at least three biological replicates. Data are presented as mean ± SEM. Group comparisons were performed using one-way ANOVA with Tukey's post-hoc test, or Student's *t*-test where appropriate. $P < 0.05$ or $P < 0.01$ was considered statistically significant. Statistical analyses were performed using GraphPad Prism (v9).

Supplementary tables

Table S1. Baseline Demographic, Clinical and Laboratory Data

Parameter	Lean control<br>N= 6<br>Mean ± S.D.	MASL<br>N= 8<br>Mean ± S.D.	MASH<br>N=8<br>Mean ± S.D.	P value
Age (yrs)	52.1 ± 4.0	49.9 ±14.1	57.4 ±10.3	n.s.
Males:females (n)	5:5	4:8	5:7	n.s.
Caucasian (%)	65	75	100	n.s.
BMI (kg/m2)	24.8 ± 2.3	37.1 ± 7.0	41.8 ±5.4	< 0.0001*
Type 2 diabetes mellitus (n)	0	4	4	n.s.^
Hypertension (n)	0	6	6	0.02*^
AST (IU/l)	24.5 ±6.2	30.8 ± 14.7	41.7 ± 17.8	<0.005**
ALT (IU/l)	26.3 ±15.3	70.1 ± 44.4	80.1 ± 16.8	<0.008**
Alk phos (IU/l)	87 ± 24	97 ± 18	101 ± 21	n.s.
Bilirubin (mg/dl)	0.2 ± 0.09	0.2 ± 0.1	0.3 ± 0.1	n.s.
Albumin (gm/dl)	4.1± 0.4	3.9 ± 0.3	4 ± 0.3	n.s.
Fasting blood sugar (mg/dl)	90 ± 9	98 ± 11	99 ± 12	n.s.
Fasting insulin (uIU/dl)	6.5 ±3	20 ± 11	22 ± 7	<0.005*
Hemoglobin A1C (%)	5.3 ± 0.6	6.2 ± 2.1	6.5 ± 1.4	n.s.
Total cholesterol (mg/dl)	195.6 ± 31.2	206.6 ± 70.7	216.0 ±28.9	n.s.
LDL-cholesterol (mg/dl)	107.4 ±19.4	109.8 ± 57.3	139.8 ± 17.8	<0.01*
HDL-cholesterol (mg/dl)	56.3 ± 14.8	43.6 ± 13.7	43.6 ± 8.8	<0.05**
Triglycerides (mg/dl)	97.7 ± 38.7	171.8 ± 81.6	211.3 ± 44.7	<0.01*
Median fibrosis stage	0.0 ± 0.0	1.1± 0.39	2.5± 0.61	<0.01**

* lean vs other groups

** MASL or MASH vs either control group

^ chi square for trend

Table S2. Primer sequences and PCR conditions

Target Gene	Primer Direction Sequence (5′→3′)	Amplicon Size (bp)
B2M	Forward: AGATGAGTATGCCTGCCGTG<br>Reverse: GCGGCATCTTCAAACCTCCA	126
ACTB	Forward: AGAGCTACGAGCTGCCTGAC<br>Reverse: AGCACTGTGTTGGCGTACAG	155
HSD17B13 (Variants A & B)	Forward: GCATGGAATAGGCAGGCAGA<br>Reverse: GTGCTGAGAAGATCGGCTGG	261 (A) / 153 (B)

Note: PCR reactions were performed using SYBR Green Master Mix under the following conditions unless otherwise stated: initial denaturation at 95 °C for 3 minutes, followed by 20 cycles of 95 °C for 15 seconds and 60 °C for 30 seconds.

Table S3. Candidate siRNA and pre-miRNA Sequences from HSD17B13 Variant B

Label	Type	Location (nt)	Sequence (5′→3′)	MFE (kcal/mol
1	siRNA-1	15–35	AGAAATCCTTCTGCTTCTGAT	-1.03
2	siRNA-2	220–240	GAAGTGGGTGATGTAACAATC	-0.60
3a	pre-miRNA-3a	280–310	CTCAGCACCAAGGATGAAGAGATTACCAAGA	-1.76
3b	pre-miRNA-3b	719–750	CGCCTCAGCGATTTTAAATCGTATGCAGAAT	-5.35

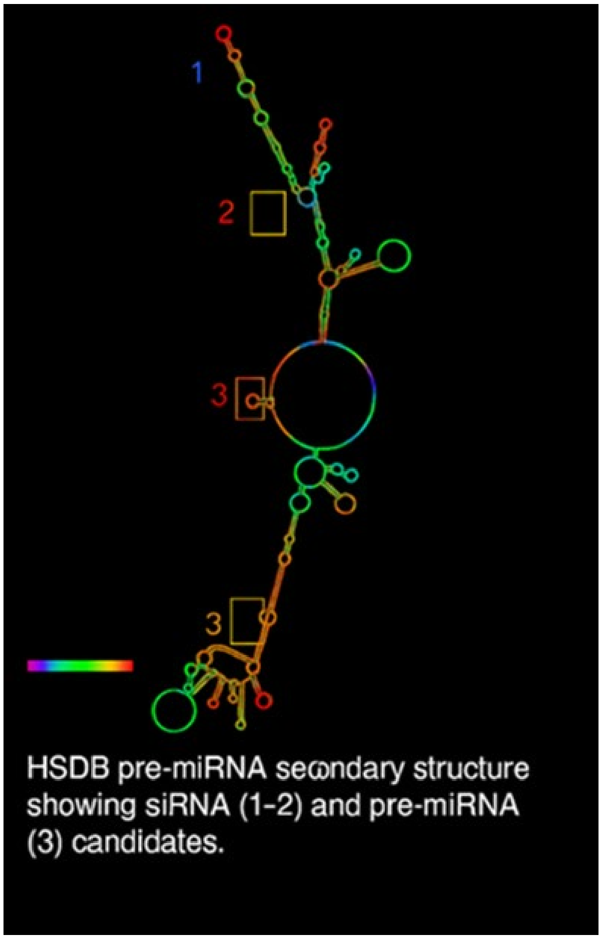

Fig. S1. Predicted RNA secondary structure and small RNA duplexes in HSD17B13 Variant B isoform.

Secondary structure modeling of full-length coding sequences (ORFs) for *HSD17B13* Variant B was performed using the ViennaRNA package RNAfold. Predicted minimum free energy (MFE) conformation is shown, with–223.80 kcal/mol for *HSD17B13* Variant B. iMultiple hairpin-loop structures and siRNA-like stem-loop duplexes (colored) were identified n HSD^{B} isoform, with three pre-miRNA–like features detected in HSD17B13 Variant B. Structural features were annotated using Biopython, highlighting potential regulatory domains. This configuration supports a model in which *HSD17B13* Variant B adopts a functional RNA secondary structure with the potential to mediate post-transcriptional silencing

Original Gels

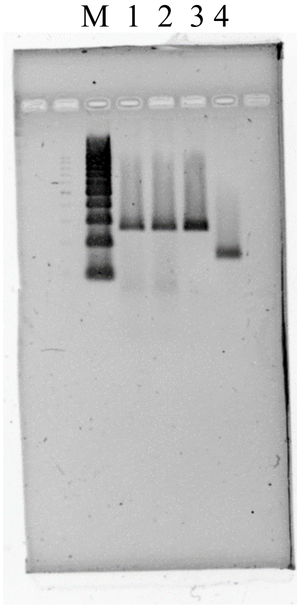

M; 100bp DNA marker

1. HepG2 cells
2. Huh-7 cells
3. HSD^{A} plasmid
4. HSD^{B} plasmid

1A

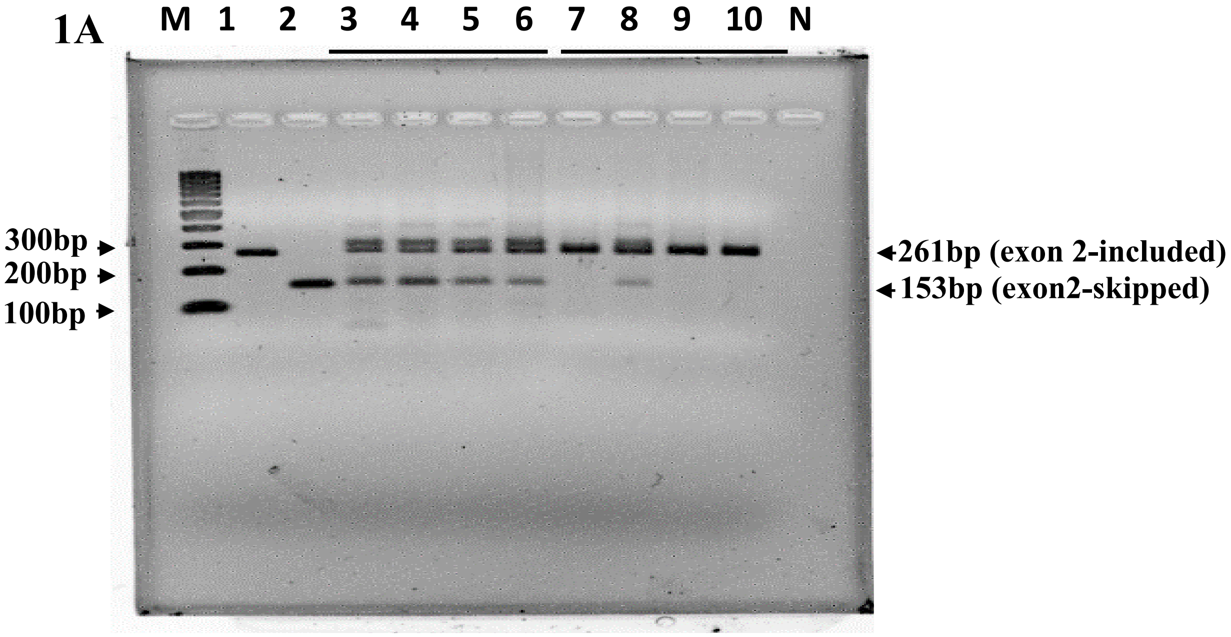

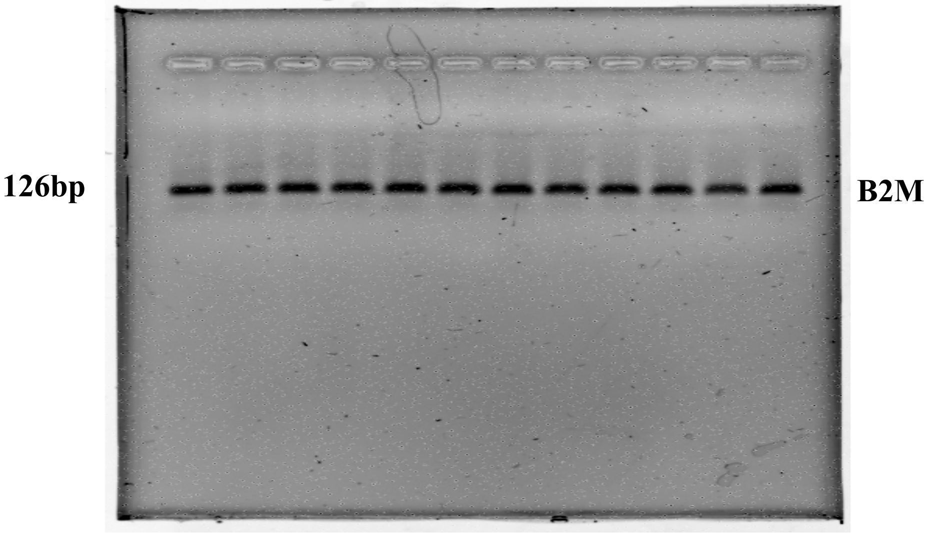

1. HepG2 cells
2. Huh-7 cells
3. Lane 3-12: Lean control, 4 samples and MASL, 6 samples

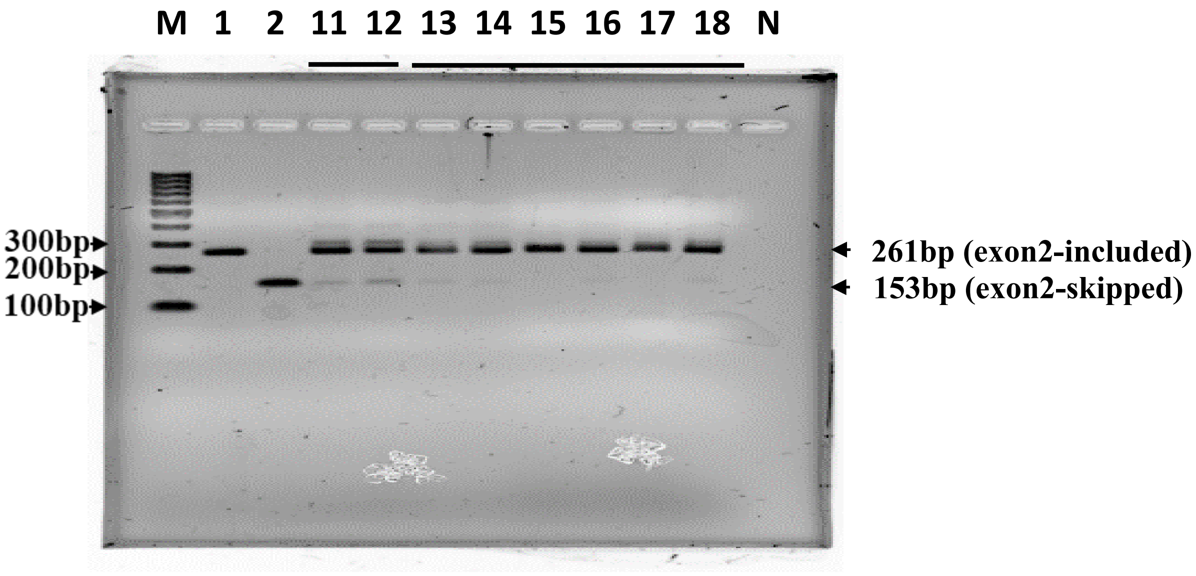

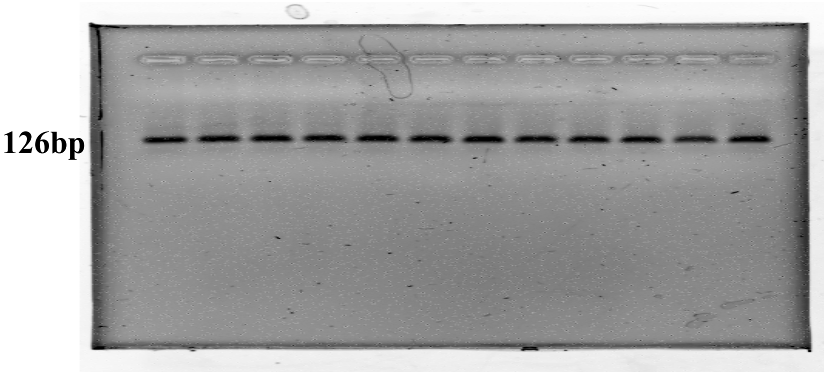

1. HepG2 cells
2. Huh-7 cells
3. Lane 3-12: MASL, 2 samples and MASH, 6 samples

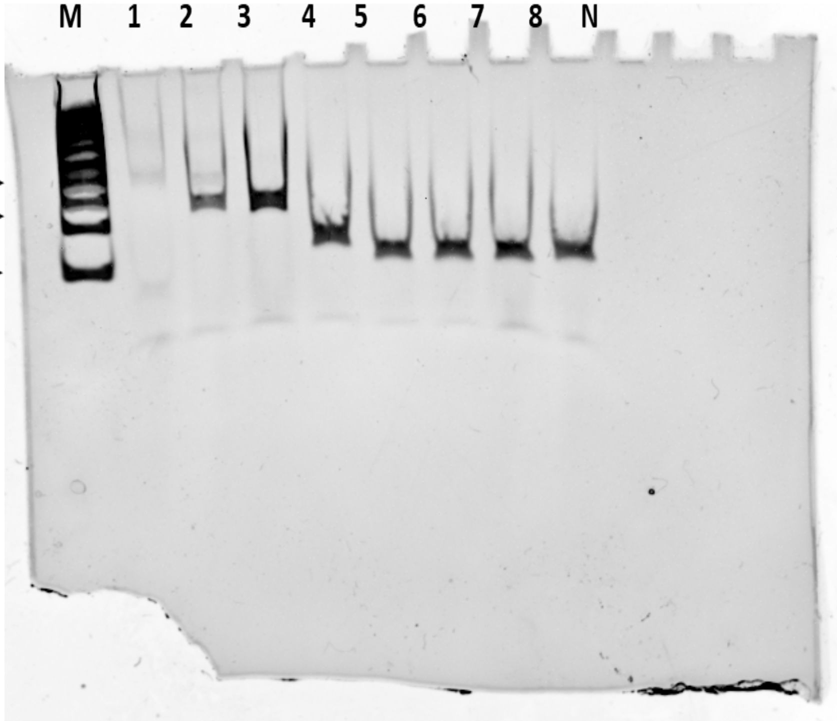

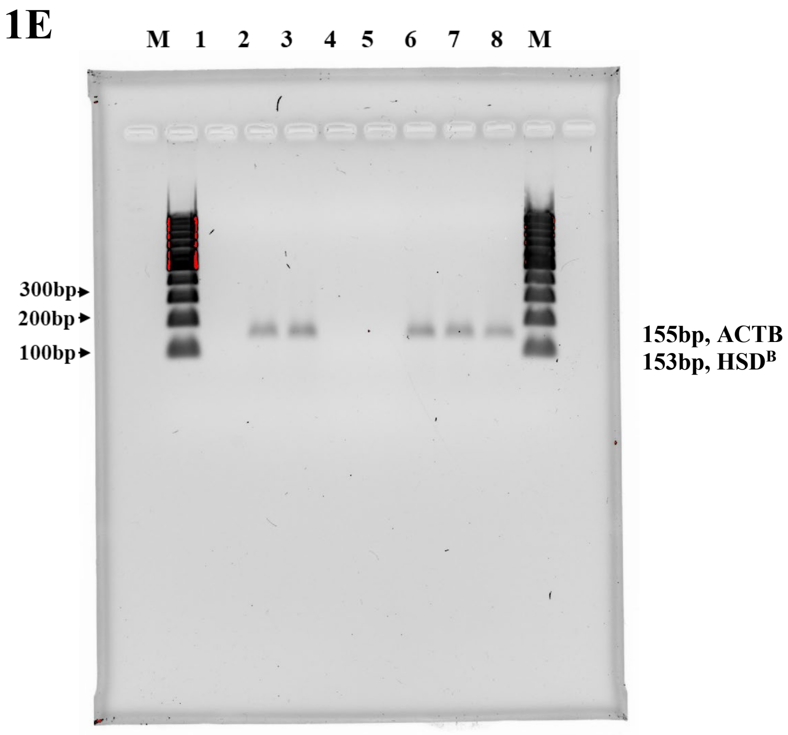
1E
M 1 2 3 4 5 6 7 8 M
300bp
200bp
100bp
155bp, ACTB
153bp, HSD^B

Immunoblot

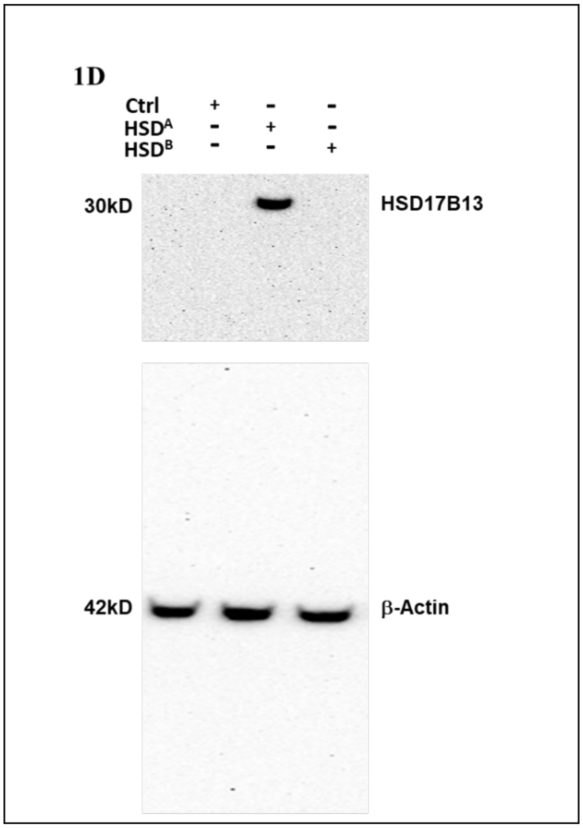
1D
Ctrl + - -
HSD^A - + -
HSD^B - - +
30kD
HSD17B13
42kD
β-Actin

Supplementary references

1. Min HK, Mirshahi F, Verdianelli A, Pacana T, Patel V, Park CG, et al. Activation of the GP130-STAT3 axis and its potential implications in nonalcoholic fatty liver disease. Am J Physiol Gastrointest Liver Physiol. 2015;308(9):G794–803.
2. Brunt EM, Janney CG, Di Bisceglie AM, Neuschwander-Tetri BA, Bacon BR. Nonalcoholic steatohepatitis: a proposal for grading and staging the histological lesions. Am J Gastroenterol. 1999;94(9):2467–74.
3. Lorenz R, Bernhart SH, Honer Zu Siederdissen C, Tafer H, Flamm C, Stadler PF, et al. ViennaRNA Package 2.0. Algorithms Mol Biol. 2011;6:26.
4. Cock PJ, Antao T, Chang JT, Chapman BA, Cox CJ, Dalke A, et al. Biopython: freely available Python tools for computational molecular biology and bioinformatics. Bioinformatics. 2009;25(11):1422–3.
5. Zuker M. Mfold web server for nucleic acid folding and hybridization prediction. Nucleic Acids Res. 2003;31(13):3406–15.
