# Supplementary material for: Disease-driven loss of inactive HSD17B13 isoforms enhances enzymatic output in MASH and counters protective rs72613567:TA variant
Source: JHEP Rep. 2026 Feb 23;8(5):101793. doi: 10.1016/j.jhepr.2026.101793 (PMC13101278; doi:10.1016/j.jhepr.2026.101793)
Supplement: Multimedia component 2 [file mmc2.docx]

**Journal of Hepatology**

**CTAT methods**

Tables for a “Complete, Transparent, Accurate and Timely account” (CTAT) are now mandatory for all revised submissions. The aim is to enhance the reproducibility of methods.

- Only include the parts relevant to your study
- Refer to the CTAT in the main text as ‘Supplementary CTAT Table’
- Do not add subheadings
- Add as many rows as needed to include all information
- Only include one item per row

**If the CTAT form is not relevant to your study, please outline the reasons why:**

| We understand that CTAT tables aim to ensure transparency and reproducibility. While our study is not clinical or interventional in nature, we have completed the CTAT form to the extent applicable. As our work involves isoform-specific gene expression, RNA structure modeling, and in vitro assays in HepG2 cells, certain clinical and patient-related sections are not relevant. |
| --- |

- 1. **Antibodies**

| **Name** | **Citation** | **Supplier** | **Cat no.** | **Clone no.** |
| --- | --- | --- | --- | --- |
| HSD17B13 | Cell Signaling Technology, #35371S | Cell signalling Co. | 35371S |  |

- 1. **Cell lines**

| **Name** | **Citation** | **Supplier** | **Cat no.** | **Passage no.** | **Authentication test method** |
| --- | --- | --- | --- | --- | --- |
| HepG2 | ATCC, HB-8065 (human hepatoma) | ATCC | HB-8065 | 5–20> | STR profiling; Mycoplasma-negative |

- 1. **Organisms**

| **Name** | **Citation** | **Supplier** | **Strain** | **Sex** | **Age** | **Overall n number** |
| --- | --- | --- | --- | --- | --- | --- |
| Not applicable | This study did not use any animals | **-** | **-** | **-** | **-** | **-** |

- 1. **Sequence based reagents**

| **Name** | **Sequence ((5′→3′))** | **Supplier** |
| --- | --- | --- |
| B2M Forward | AGATGAGTATGCCTGCCGTG | IDT / Thermo |
| B2M Reverse | GCGGCATCTTCAAACCTCCA | IDT / Thermo |
| ACTB Forward | AGAGCTACGAGCTGCCTGAC | IDT / Thermo |
| ACTB Reverse | AGCACTGTGTTGGCGTACAG | IDT / Thermo |
| HSD17B13 Forward | GCATGGAATAGGCAGGCAGA | IDT / Thermo |
| HSD17B13 Reverse | GTGCTGAGAAGATCGGCTGG | IDT / Thermo |

- 1. **Biological samples**

| **Description** | **Source** | **Identifier** |
| --- | --- | --- |
| Human liver tissue (lean control) | Virginia Commonwealth University (VCU) Biorepository | Cohort A, n = 6 |
| Human liver tissue (MASLD without MASH) | VCU Biorepository | Cohort B, n = 8 |
| Human liver tissue (MASH) | VCU Biorepository | Cohort C, n = 8 |

- 1. **Deposited data**

| **Name of repository** | **Identifier** | **Link** |
| --- | --- | --- |
| Not applicable | **-** | **-** |

- 1. **Software**

| **Software name** | **Manufacturer** | **Version** |
| --- | --- | --- |
| ImageJ (FIJI) | NIH (U.S. Government) | 2.14.0 |
| RNAfold | ViennaRNA Websuite | 2.5.1 (Webserver) |
| Biopython | Open-source | 1.81 |
| GraphPad Prism | GraphPad Software | 10.1.0 |
| Adobe Illustrator | Adobe Inc. | 28.0 |
| Microsoft Excel | Microsoft Corporation | Office 365 |

- 1. **Other (e.g. drugs, proteins, vectors etc.)**

| **Name** | **Description** | **Supplier** |
| --- | --- | --- |
| Oleic acid | Lipogenic stress inducer (used at 400 μM in HepG2 cells) | Sigma-Aldrich |
| Plasmid: Variant A | HSD17B13 full-length (exon 2-included) overexpression vector | Origene Co. |
| Plasmid: Variant B | HSD17B13 exon 2-skipped isoform (non-enzymatic) | Origene Co. |
| RNase III | dsRNA-specific endoribonuclease | New England Biolabs (NEB) |
| RNase A | ssRNA endoribonuclease | ThermoFisher Scientific |
| RNase T1 | ssRNA guanine-specific ribonuclease | ThermoFisher Scientific |

- 1. **Please provide the details of the corresponding methods author for the manuscript:**

| **Full Name** | **Institution** | **Email** |
| --- | --- | --- |
| Arun J. Sanyal | VCU | arun.sanyal@vcuhealth.org |
| Hae-Ki Min | VCU | hae-ki.min@vcuhealth.org |

**2.0 Please confirm for randomised controlled trials all versions of the clinical protocol are included in the submission. These will be published online as supplementary information.**

| Not applicable |
| --- |
